# First characterization of four repeat regions with the *bla*_NDM-1_ carried on an IncFII plasmid in *Enterobacter hormaechei*

**DOI:** 10.1016/j.isci.2025.112369

**Published:** 2025-04-08

**Authors:** Yi Liu, Xiaojing Liu, Ruishan Liu, Hao Xu, Mantao Chen, Jiajie Qian, Beiwen Zheng

**Affiliations:** 1 State Key Laboratory for Diagnosis and Treatment of Infectious Diseases, Collaborative Innovation Center for Diagnosis and Treatment of Infectious Diseases, the First Affiliated Hospital, Zhejiang University School of Medicine, Hangzhou, China; 2 Shandong First Medical University and Shandong Academy of Medical Sciences, Jinan, China; 3 Department of Structure and Morphology, Jinan Microecological Biomedicine Shandong Laboratory, Jinan, Shandong, China; 4 Department of Neurosurgery, the First Affiliated Hospital, College of Medicine, Zhejiang University, Hangzhou, China; 5 Department of Gastrointestinal Surgery, The First Affiliated Hospital, College of Medicine, Zhejiang University, Hangzhou, China; 6 Research Units of Infectious Diseases and Microecology, Chinese Academy of Medical Sciences, Beijing, China; 7 Yuhang Institute for Collaborative Innovation and Translational Research in Life Sciences and Technology, Hanghzou, China

**Keywords:** Molecular biology, Microbiology

## Abstract

Carbapenem-resistant Enterobacteriaceae (CRE) have emerged as a critical threat to global health, and multicopy resistance genes are a potential contributor to increased resistance. This study investigates a novel arrangement of four *bla*_NDM-1_ genes in tandem on an IncFII plasmid from *Enterobacter hormaechei*. Whole-genome sequencing (WGS) of *E. hormaechei* isolates L710hy identified a four-tandem repeat of the *bla*_NDM-1_ gene, with each copy’s environment nearly identical (IS*CR1*-*aph(3′)-VI*-IS*Aba125*-*bla*_NDM-1_-*ble*-*iso*-*tat*-*dsbD*-*cutA*-*sul1*-IS*CR1*). The study suggests a correlation between the *bla*_NDM-1_ copy number and minimum inhibitory concentration (MIC) values for carbapenems, though further research is needed to confirm this correlation. The stability of these four *bla*_NDM-1_ tandem repeat structures was also investigated. This is the first documented case of *bla*_NDM-1_ tandem repeats in *E. hormaechei*, and the IS*CR1* and other insertion sequence may play a role in this arrangement.

## Highlights

• Identification of four tandem *bla*_NDM-1_ copies in *Enterobacter hormaechei*
• The insertion sequence may mediate the occurrence of multicopy *bla*_NDM-1_
• Tandem repeats of four *bla*_NDM-1_ exhibit instability in the absence of antibiotic pressure

## Graphical abstract

**Figure:**
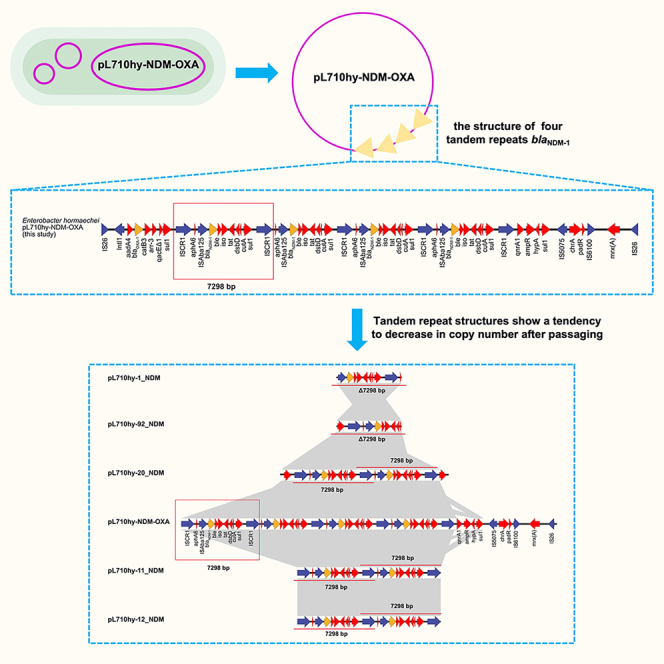

## Introduction

The *Enterobacter cloacae complex* (ECC) has emerged as the third most prevalent drug-resistant Enterobacteriaceae causing nosocomial infections, following *Escherichia coli* and *Klebsiella pneumoniae*. It has notably become a formidable pathogen, posing a threat to global public health.^1^ *E. hormaechei* is commonly observed in clinical samples. ECC exhibits an increased production of AmpC β-lactamase through the acquisition of a transferable *ampC* gene on a plasmid or mobile element, resulting in resistance to third-generation cephalosporins.^2^

Several New Delhi metallo-beta-lactamase(NDM)variants have been detected in *E. hormaechei*. Among them, *bla*_NDM-1_ has been detected worldwide with the highest detection rate,^3^^,^^4^^,^^5^ *bla*_NDM-7_ was detected from Canada and Spain,^6^ and *bla*_NDM-5_ was detected in urine samples from a diabetic patient in China.^7^ Insertion sequences and resistance genes are strongly correlated.^8^ The association of *bla*_NDM-1_ and Tn*125* was initially described in *Acinetobacter baumannii*.^9^ The surrounding environment of resistance genes plays an important role in their expression as well as the resistance phenotype. Insertion sequences located upstream of resistance genes can act as promoters, enhancing gene expression. Inserted sequence (IS)*Aba125* is often found upstream of *bla*_NDM-1_, functioning as a promoter to regulate its expression.

Single-copy *bla*_NDM-1_ is commonly detected in *E. hormaechei*, while reports of multicopy *bla*_NDM-1_ remain rare. Antimicrobial susceptibility testing (AST) revealed that *E. hormaechei* L710hy harboring multicopy *bla*_NDM-1_ exhibited a higher level of resistance to imipenem and meropenem. The genetic environment of *bla*_NDM-1_ was found to contain multiple insertion elements, which may mediate the generation of multicopy *bla*_NDM-1_ regions. Previous studies have already shown that IS*CR1*, IS*Aba125*, and Tn*3* are associated with the appearance of multicopy *bla*_NDM-1_.^10^^,^^11^ In this study, we characterized a novel arrangement of four *bla*_NDM-1_ genes arranged in tandem on an IncFII plasmid from *E. hormaechei* isolate L710hy. We performed genome sequencing, AST, S1-PFGE, conjugation assays, and stability assays to explore the resistance profile and plasmid characterization of L710hy and stability of tandem repeat regions harboring *bla*_NDM-1_, while proposing a hypothesis on insertion elements in mediating the generation of multicopy *bla*_NDM-1_.

## Results

### AST

L710hy showed resistance to aztreonam, imipenem, meropenem, ceftriaxone, cefotaxime, ceftazidime, levofloxacin, ciprofloxacin, amikacin, gentamicin, piperacillin-tazobactam, and cefepime, intermediate susceptibility to polymyxin B, and sensitivity to tigecycline, chloramphenicol, and trimethoprim-sulfamethoxazole (Table 1).

**Table 1:** Minimum inhibitory concentrations of *E. hormaechei* L710hy, recipient strain PAO1Ri, transconjugants L710hy-PAO1Ri, and control strain ATCC 25922

Antimicrobials	L710hy	L710hy-PAO1Ri	PAO1Ri	ATCC 25922
Aztreonam	128 (R)^a^	128 (R)	2 (S)	0.25 (S)
Imipenem	64 (R)	64 (R)	4 (I)	0.06 (S)
Meropenem	8 (R)	8 (R)	2 (S)	0.03 (S)
Ceftriaxone	>128 (R)	>128 (NA)	4 (NA)	0.03 (S)
Cefotaxime	>128 (R)	>128 (R)	8 (R)	0.5 (S)
Ceftazidime	>128 (R)	>128 (R)	1 (S)	0.25 (S)
Levofloxacin	64 (R)	64 (R)	0.25 (S)	0.03 (S)
Ciprofloxacin	>64 (R)	>64 (R)	0.5 (S)	0.004 (S)
Chloramphenicol	8 (S)	64 (NA)	64 (NA)	8 (S)
Trimethoprim-sulfamethoxazole	0.25/4.75 (S)	>8/152 (NA)	>8/152 (NA)	0.125/2.375 (S)
Amikacin	>128 (R)	>128 (R)	2 (S)	4 (S)
Gentamicin	>128 (R)	>128 (R)	32 (R)	2 (S)
Piperacillin-tazobactam	128/4 (R)	>128/4 (R)	4/4 (S)	4/4 (S)
Cefepime	64 (R)	32 (R)	1 (S)	0.125 (S)
Polymyxin B	0.06 (I)	0.03 (S)	0.03 (S)	0.06 (S)
Tigecycline	0.03 (S)	0.03 (NA)	0.03 (NA)	0.03 (S)

a SIR, susceptible (S), intermediate (I), resistant (R), not applicable (NA).

### Plasmid analysis and conjugation assay

**Figure 1:**
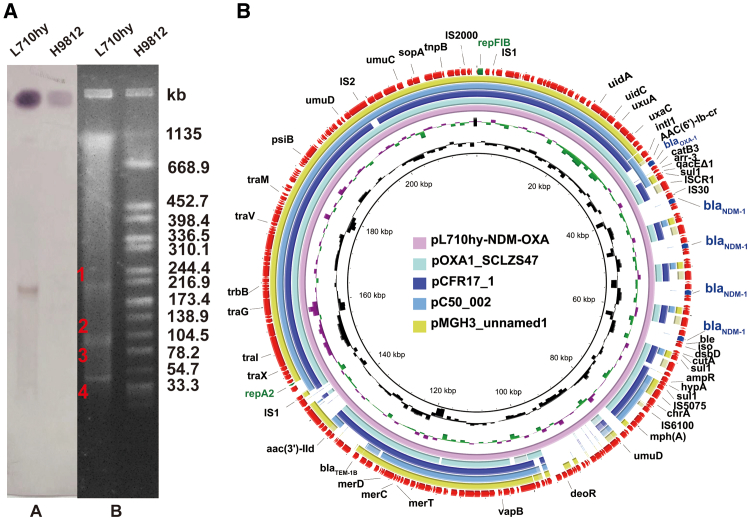
Characterization of plasmids pL710hy-NDM-OXA (A) Plasmid characteristic of *E. hormaechei* L710hy. Southern blotting hybridization with *bla*_NDM-1_-specific probe is in region A. Plasmid size determination by S1-PFGE is in region B, with the marker *Salmonella enterica* serotype Braenderup H9812. (B) Sequence comparison analysis of pL710hy-NDM-OXA with pCFR17_1 (GenBank: CP035277), pOXA1_SCLZS47 (GenBank: CP092496), pC50_002 (GenBank: CP042480), and pMGH3_unnamed1 (GenBank: CP072954). Replication genes (*repA2* and *repFIB*), antibiotic resistance genes (ARGs), and other elements are highlighted in green, blue, and red, respectively.

S1-PFGE showed that L710hy contained four plasmids, and southern blotting showed that *bla*_NDM-1_ was present in the 218,724 bp plasmid pL710hy-NDM-OXA (Figure 1A). pL710hy-NDM-OXA was identified as an IncFII (Yp) plasmid, with a GC content of 53.6%. According to the NCBI BLAST search results, plasmid pL710hy-NDM-OXA was almost identical to pCFR17_1 (GenBank: CP035277), pOXA1_SCLZS47 (GenBank: CP092496), pC50_002 (GenBank: CP042480), and pMGH3_unnamed1 (GenBank: CP072954), with similarities of more than 99% (Figure 1B).

We evaluated the transferability of the plasmid pL710hy-NDM-OXA through conjugation assays and found it could be successfully transferred to the recipient strain *Pseudomonas aeruginosa* PAO1Ri as verified by MALDI-TOF/mass spectrometry (MS) and PCR. The transconjugants (L710hy-PAO1Ri) carrying *bla*_NDM-1_ showed resistance to aztreonam, imipenem, meropenem, cefotaxime, ceftazidime, levofloxacin, ciprofloxacin, amikacin, gentamicin, piperacillin-tazobactam, and cefepime, while remaining susceptible to polymyxin B (Table 1). Notably, the minimum inhibitory concentration (MIC) values for chloramphenicol and trimethoprim-sulfamethoxazole in the transconjugants were higher than those in L710hy, whereas the MIC values for the other antibiotics were similar between the transconjugants and L710hy.

### Genomic characterization of isolate L710hy

The genome of isolate L710hy contained a 4,975,189 bp circular chromosome, with GC content of 55.2%, and four plasmids between 43,256 to 218,724 bp in size. L710hy was identified as ST93 by MLST (*dnaA*(9), *fusA*(4), *gyrB*(14), *leuS*(61), *pyrG*(37), *rplB*(4), *rpoB*(9)). Sequencing results showed that plasmid pL710hy-NDM-OXA carries *qacEΔ1*, *arr-3*, *aac(6′)-lb-cr*, *aac(3)-lld*, *mph(A)*, *bla*_TEM-1B_, *bla*_OXA-1_, *qnrA1*, *catB3*, *sul1*, and four tandem repeats of *bla*_NDM-1_. Comprehensive information regarding the chromosomes and plasmids of L710hy is presented in Table 2.

**Table 2:** Features of chromosome and plasmids harbored by *E. hormaechei* L710hy

Name	Size (bp)	ST	Plasmid type	Antimicrobial resistance genes
chromosome	4,975,189	93	–	*fosA*, *bla*_ACT-7_, *bla*_CTX-M-14_
pL710hy-NDM-OXA	218,724	–	IncFII(Yp)	*qacEΔ1*, *arr-3*, *aac(6′)-lb-cr*, *aac(3)-lld*, *mph(A)*, *bla*_TEM-1B_, *bla*_NDM-1_, *bla*_OXA-1_, *qnrA1*, *catB3*, *sul1*
pL710hy-2	112,363	–	IncFIB(pHCM2)	–
pL710hy-3	87,782	–	IncFII(Yp)	*qacEΔ1*, *armA*, *aadA1*, *mph(E)*, *msr(E)*, *bla*_TEM-1B_, *sul1*
pL710hy-4	43,256	–	–	–

**Figure 2:**
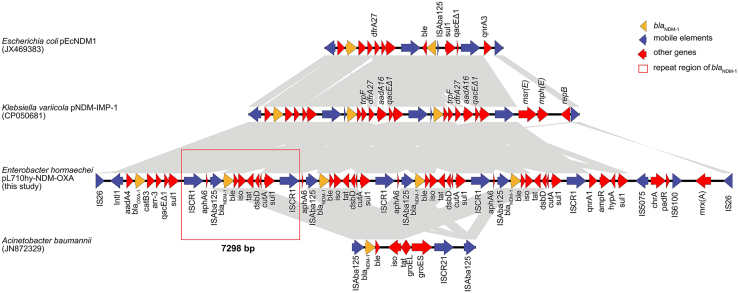
The genetic context of *bla*_NDM-1_ Comparison of genes surrounding *bla*_NDM-1_ on pL710hy-NDM-OXA, pEcNDM1, pNDM-IMP-1, and Tn*125* in *Acinetobacter baumannii.* Open Reading Frames (ORFs) are shown as arrows and indicated according to their putative functions. Yellow indicates resistance gene *bla*_NDM-1_, blue indicates genes related to mobile elements, red represents other functional genes, and red box indicates 7,298 bp repeat unit of *bla*_NDM-1_. Regions with a high degree of homology are indicated by gray shading.

The genetic environment of each *bla*_NDM-1_ forms a structure like Tn*125* (GenBank: JN872329)^9^ but distinctively exhibits insertions of *aphA6* and *dsbD-cutA-sul1*, deletions of *groEL* and *groES*, and a substitution of IS*CR21* for IS*CR1*. In pL710hy-NDM-OXA, the upstream of the IS*CR1* is marked by *intl1* and six resistance gene cassettes (*aadA4-bla*_OXA-1_*-catB3-aar-3-qacED1-sul1*), consistent with the *int1*-IS*CR1* structure observed in pNDM-IMP-1 (GenBank: CP050681). Compared to *E. coli* pEcNDM1 (GenBank: JX469383), pL710hy-NDM-OXA does not contain *dfrA27* (Figure 2). In pL710hy-NDM-OXA, four copies of *bla*_NDM-1_ were found in four tandemly linked 7,298 bp regions. Within each 7,289 bp region, IS*CR1* and IS*Aba125* appear upstream of *bla*_NDM-1_. Meanwhile, IS*26* was found upstream and downstream of the first and last *bla*_NDM-1_, respectively. Also, IS*5075* and IS*6100* were found downstream of the last *bla*_NDM-1_ (Figure 2).

### Stability of the *bla*_NDM-1_ structure with four tandem repeats

**Figure 3:**
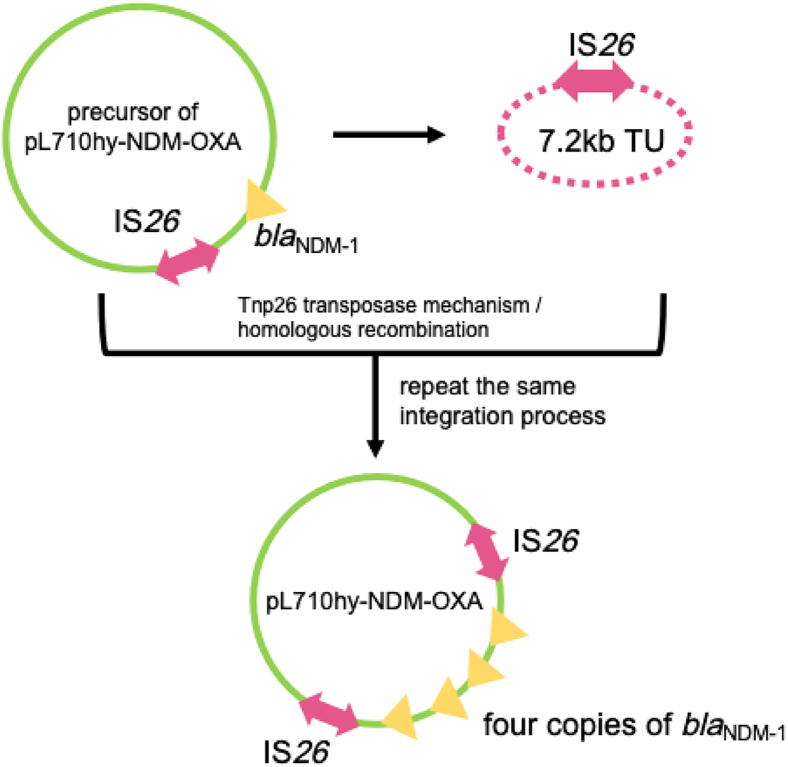
Putative mechanisms of the formation of the four *bla*_NDM-1_ tandem repeats

Strain L710hy was passaged, and a 50th generation suspension was obtained. After dilution and plating, 150 single clones were isolated for further analysis. MALDI-TOF/MS identified all clones as *E. hormaechei*, and PCR confirmed the presence of the *bla*_NDM-1_ gene in all isolates. To investigate the stability of the four-tandem *bla*_NDM-1_ arrangement, five clones (L710hy-1, L710hy-92, L710hy-11, L710hy-12, and L710hy-20) were selected randomly for nanopore sequencing analysis. The results showed that L710hy-1 and L710hy-92 each carried one copy of the *bla*_NDM-1_ gene on a 7,703 bp plasmid, L710hy-11 and L710hy-12 each carried two copies on a 203,318 bp plasmid, and L710hy-20 contained two copies on a 103,188 bp plasmid. BLAST analysis revealed that all five plasmids shared over 99% sequence identity with pL710hy-NDM-OXA (218,724 bp). In addition, the genetic environment of *bla*_NDM-1_ in these plasmids was almost identical to that of pL710hy-NDM-OXA (Figure 3). The structure of the four-tandem repeat *bla*_NDM-1_ undergoes noticeable alterations during passaging, as reflected by a reduction in *bla*_NDM-1_ copy numbers. However, the genetic environment surrounding *bla*_NDM-1_ remains unchanged in each strain obtained through the passage process.

## Discussion

*E. hormaechei* is among the predominant species in ECC causing nosocomial infections. It has been reported to be associated with several nosocomial outbreaks and has emerged as an important pathogen causing clinical infections in humans.^6^^,^^12^^,^^13^^,^^14^ The existence and prevalence of *E. hormaechei* is a potentially great threat to human health. Multiple copies of drug-resistant genes may increase the burden of carbapenem resistance. Therefore, the mechanism of the occurrence of multicopy drug-resistant genes should be focused.

Previous studies have predominantly reported the presence of a single-copy *bla*_NDM-1_ gene encoded by various plasmids. However, in recent years, there have been a growing number of reports highlighting the emergence of multicopy *bla*_NDM-1_ scenarios.^10^^,^^15^ Notably, within pL710hy-NDM-OXA, four copies of the *bla*_NDM-1_ were identified, located in four tandemly connected 7,298 bp regions. Further analysis of the 7,289 bp repeat region containing *bla*_NDM-1_ showed multiple insertion sequences within and around this repeat region, such as IS*CR1*, IS*Aba125*, IS*26*, IS*5075*, and IS*6100*. Taken together, these results suggest that insertion elements may play an important role in the generation of multiple repeat sequences of *bla*_NDM-1_.^8^ The amplification of resistance genes is a novel mechanism of antibiotic resistance, posing a significant challenge to the effectiveness of antibiotic treatments.^16^ Generally, the resistance conferred by gene amplification is temporary and tends to disappear in the absence of antibiotic selection pressure. During passaging without antibiotic pressure, the four tandem repeat structures of *bla*_NDM-1_ in this study showed a loss of 2 or 3 copies of *bla*_NDM-1_, which can be attributed to homologous recombination between sister chromatids.^17^ Among these, the *bla*_NDM-1_ genes in L710hy-1 and L710hy-92 were located on a small plasmid, which could be caused by transposition.

**Figure 4:**
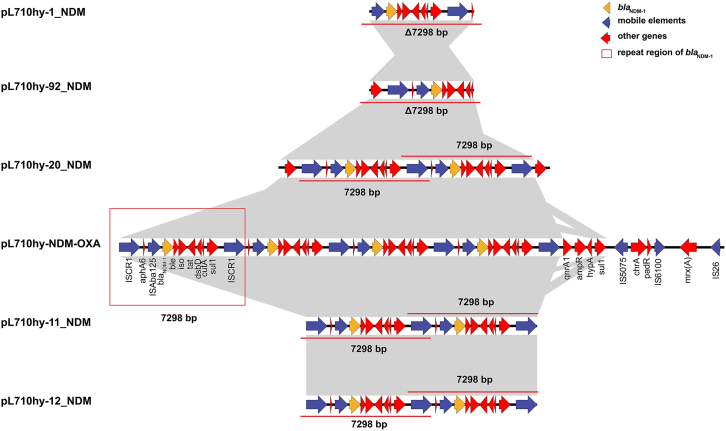
Comparison of the genetic environments of *bla*_NDM-1_ carried by pL710hy-NDM-OXA, L710hy-1, L710hy-11, L710hy-12, L710hy-20, and L710hy-92 ORFs are shown as arrows and labeled according to their putative function. Yellow indicates the resistance gene *bla*_NDM-1_, blue indicates genes associated with mobile elements, red indicates other functional genes, and red boxes indicate the 7,298 bp repeating unit of *bla*_NDM-1_. Regions with high homology are shaded in gray.

IS*26* plays a key role in the transmission of resistance genes, demonstrating two distinct modes of IS*26* movement: (1) replication of IS*26* and (2) merging of the translocatable unit (TU) with IS*26*.^18^^,^^19^ The TU consists of a DNA fragment carrying a resistance gene and an IS copy, leading to the formation of arrays of resistance genes upon repetition of the process. In a novel IS*26* movement pattern, the frequency of cointegrate formation of these two parts is significantly increased when IS*26* recognizes IS*26* in another target plasmid during transposition.^19^ These findings provide a possibility for the new arrangement that has emerged in this study: four tandem *bla*_NDM-1_ regions. IS*26* surrounds the four *bla*_NDM-1_ repeat regions, and we speculate that the existing IS*26* is incorporated into the 7.2 kb TUs through either the Tnp26 transposase-catalyzed mechanism or homologous recombination. The tandem repeat structure of *bla*_NDM-1_ is the result of several TU integration processes (Figure 4). However, this is a conjecture based on existing studies, and we cannot be sure that the four repeat regions of *bla*_NDM-1_ are those generated by IS*26* and not by other insertion elements, the mechanism of which needs to be elucidated by more detailed studies in future.

According to the findings of the current study, no definitive conclusion can be drawn regarding the impact of the *bla*_NDM-1_ copy number on carbapenem resistance. Previous reports have presented conflicting evidence, indicating that multiple copies of *bla*_NDM-1_ can either result in heightened carbapenem resistance^20^ or have no discernible effect,^10^^,^^21^ both of which have been documented. *E. hormaechei* strain L710hy harboring four *bla*_NDM-1_ showed a high level of resistance to meropenem and imipenem. However, it was still not possible to make conclusions about the *bla*_NDM-1_ copy number on carbapenem resistance in this study. Specific conclusions can only be made by comparing the resistance of L710hy with an isogenic strain that carries only a single *bla*_NDM-1_, but obtaining an isogenic strain that carries a single copy of *bla*_NDM-1_ is difficult. Moreover, a comprehensive review of antibiotic resistance in *E. hormaechei* showed that strains carrying the *bla*_NDM-1_ had an MIC value of 64 mg/L for imipenem and 32 mg/L for meropenem. Nevertheless, the specific copy number of *bla*_NDM-1_ in that strain remained undetermined.^22^ Further investigations are warranted to elucidate the precise mechanism through which gene duplication influences carbapenem resistance.

This study marks the documentation of four tandem repeats of *bla*_NDM-1_ located on an IncFII plasmid. A detailed analysis of the plasmid characteristics and the surrounding genetic environment of the repeat sequences is also presented, and the possibility of the role of insertion sequences in the formation of the *bla*_NDM-1_ tandem repeat sequences is raised, while also investigating the stability of this tandem repeat structure. In addition, the present study identified the enhancement of antibiotic resistance by multicopy resistance genes, but more detailed studies need to be conducted in the future to explore the mechanisms involved.

### Limitations of the study

Four *bla*_NDM-1_ tandem repeat regions were characterized using a strain of *E. hormaechei* of clinical origin. One of the limitations of this study is that only one strain was involved, but the paper focuses on the characterization of the tandem regions. The proposed mediating effect of insertion elements on the generation of multicopy resistance genes and the effect of multicopy resistance genes on antibiotic resistance in the paper are only inferences based on the L710hy genome characterization and AST results, which need to be used in future work to reveal the mechanisms involved.

## Resource availability

### Lead contact

Further information or requests can be directed to the lead contact, Beiwen Zheng (zhengbw@zju.edu.cn).

### Materials availability

• This study did not generate new unique reagents.
• There are restrictions to the availability of the *E. hormaechei* clinical isolate L710hy used in this study because it was stored in the hospital system that restricts distribution of the bacteria, but we can guide you to the proper contact.

### Data and code availability

• The genome of *E. hormaechei* clinical isolate L710hy has been deposited at NCBI database (BioProject ID: PRJNA932578) and is publicly available as of the date of publication. Accession numbers are listed in the key resources table. Nanopore sequencing data of strain L710hy-1, L710hy-11, L710hy-12, L710hy-20, and L710hy-92 have been deposited at Mendeley Data and are publicly available as of the date of publication (https://data.mendeley.com/datasets/czm58fp6g7/1). DOI numbers are listed in the key resources table.
• This paper does not report original code.
• This paper does not report any other items.

## Acknowledgments

This work was supported by the National Key R&D Program of China (2020YFE0204300 and 2023YFC2308400), 10.13039/501100001809National Natural Science Foundation of China (82072314), 10.13039/501100004731Zhejiang Provincial Natural Science Foundation of China (LHDMZ22H190002 and LY19H160060), Shandong Provincial Laboratory Project (SYS202202), Research Project of Jinan Microecological Biomedicine Shandong Laboratory (JNL-2022011B), Fundamental Research Funds for the Central Universities (2022ZFJH003), and CAMS Innovation Fund for Medical Sciences (2019-I2M-5-045).

## Author contributions

Study design: H.X., R.S.L., and M.T.C.; data collection: Y.L., X.J.L.; data analysis: H.X., R.S.L.; writing: Y.L.; revision of manuscript: J.J.Q. and B.W.Z. All authors reviewed the manuscript.

## Declaration of interests

The authors declare no competing interests.

## STAR★Methods

### Key resources table

**Table:** 

REAGENT or RESOURCE	SOURCE	IDENTIFIER
**Bacterial and virus strains**		

*Enterobacter hormaechei* L710hy	This study	L710hy
ATCC 25922	Laboratory stock	ATCC 25922
*Salmonella Braenderup* serotype strain (H9812)	Laboratory stock	H9812
*Pseudomonas aeruginosa* PAO1Ri	Laboratory stock	PAO1Ri
*Enterobacter hormaechei* L710hy-1	This study	L710hy-1
*Enterobacter hormaechei* L710hy-11	This study	L710hy-11
*Enterobacter hormaechei* L710hy-12	This study	L710hy-12
*Enterobacter hormaechei* L710hy-20	This study	L710hy-20
*Enterobacter hormaechei* L710hy-92	This study	L710hy-92

**Chemicals, peptides, and recombinant proteins**		

LB Broth	Solarbio	Cat# L8291
Mueller-Hinton Agar	OXOID	Cat# CM0337B

**Deposited data**		

Sequence of L710hy	This study	BioProject: PRJNA932578
Sequence of L710hy-1	This study	Mendeley Data: https://doi.org/10.17632/czm58fp6g7.1
Sequence of L710hy-11	This study	Mendeley Data: https://doi.org/10.17632/czm58fp6g7.1
Sequence of L710hy-12	This study	Mendeley Data: https://doi.org/10.17632/czm58fp6g7.1
Sequence of L710hy-20	This study	Mendeley Data: https://doi.org/10.17632/czm58fp6g7.1
Sequence of L710hy-92	This study	Mendeley Data: https://doi.org/10.17632/czm58fp6g7.1

**Software and algorithms**		

Unicycler software	Wick et al.^23^	Unicycler
PlasmidFinder	Carattoli, A. et al.^24^	PlasmidFinder
BRIG	Alikhan et al.^25^	BRIG
RAST	Aziz et al.^26^	RAST
ISfinder	Siguier et al.^27^	ISfinder
Easyfig	Sullivan et al.^28^	Easyfig
ResFinder	http://genepi.food.dtu.dk/resfinder	ResFinder

### Experimental model and study participant details

#### Bacterial strain

In May 2017, strain L710hy was isolated from the fecal sample of a 71-year-old female patient with pneumonia at the Department of Infectious Diseases, First Affiliated Hospital of Zhejiang University School of Medicine. The strain was identified as Enterobacter hormaechei by WGS.L710hy was found to have four tandem sequences of *bla*_NDM-1_ in a single plasmid, and to investigate the stability of the tandem sequences. To investigate the stability of the tandem sequences, several single colonies were obtained after passaging and five of these (L710hy-1, L710hy-11, L710hy-12, L710hy-20, and L710hy-92) were subjected to nanopore sequencing.

#### Ethics approval and consent to participate

The study was approved by the Ethics Committee of the First Affiliated Hospital of Zhejiang University School of Medicine (reference number: 2018-752). This was a retrospective study and patient consent was not required.

### Method details

#### Clinical isolates and determination of antibiotic resistance genes

In May 2017, strain L710hy was isolated from fecal samples obtained from a 71-year-old female patient with pneumonia in the Department of Infectious Diseases, the First Affiliated Hospital of Zhejiang University School of Medicine. Identification of the strains and resistance genes was accomplished through the MALDI-TOF/MS (Bruker, Bremen, Germany). We depended on PCR to identify common carbapenemase-encoding genes. Primer sequences are as follows^29^: *bla*_NDM_ (F: ATGGAATTGCCCAATATTATGCAC; R: TCAGCGCAGCTTGTCGGC), *bla*_KPC_ (F: ATGTCACTGTATCGCCGTC; R: TTACTGCCCGTTGACGCC), *bla*_IMP_ (F: GTTTATGTTCATACWTCG; R: GGTTTAAYAAAACAACCAC), *bla*_VIM_ (F: TTTGGTCGCATATCGCAACG; R: CCATTCAGCCAGATCGGCAT) and *bla*_OXA-48_ (F: TTGGTGGCATCGATTATCGG; R: GAGCACTTCTTTTGTGATGGC).

#### Antimicrobial susceptibility testing (AST)

The AST of L710hy for tigecycline and polymyxin B was performed by broth microdilution methods. For all other antibiotics, AST was performed by the agar dilution method. *Escherichia coli* ATCC 25922 was used as a control strain. These results were interpreted according to the Clinical Laboratory Standards Institute CLSI^30^ and the European Committee on Antimicrobial Susceptibility Testing EUCAST^31^ guidelines.

#### S1 pulsed-field gel electrophoresis (S1-PFGE)

S1-PFGE indicates the size and number of plasmids.^32^ Southern blotting indicates the plasmid where *bla*_NDM-1_ is located. S1-PFGE was performed on a contour-clamped homogeneous electric field (CHEF) technique (Bio-Rad. Hercules, CA, United States). Experimental methods were modified from previously published guidelines.^33^ Electrophoresis was performed for 16 h with pulse time from 2.16s to 6.38 s at 5–500 k and 14°C. The Xba-I digested *Salmonella Braenderup* serotype strain (H9812) was used as the DNA size marker. 4 MacFarland (McF) bacterial suspensions were mixed with 1% Seakem Golden agarose and 1% sodium dodecyl sulfate (SDS) and digested with proteinase K at 54°C for 2 h, followed by digestion with S1 nuclease. We then used a digoxigenin-labeled *bla*_NDM-1_ probe made from dig-high prime DNA Labeling and Detection Starter Kit II (Roche Diagnostics, Switzerland) to identify the location of the plasmid carrying *bla*_NDM-1_ by Southern blotting.

#### Conjugation assay

Rifampicin-resistant *Pseudomonas aeruginosa* PAO1Ri was used as a recipient strain to test plasmid transferability. PAO1Ri and L710hy were separately incubated in 5 mL of LB broth with shaking for 6 h at 37°C, mixed proportionally, and then incubated for 24 h at 37°C. Transconjugants were screened on Mueller-Hinton (OXOID, Hampshire, United Kingdom) medium with 200 mg/L rifampicin and 2 mg/L meropenem and verified by MALDI-TOF/MS and PCR.

#### Stability assays of tandem arrangements

Strain L710hy was cultured in LB broth at 37°C with shaking at 180 rpm and subsequently passaged at a 1:1000 dilution in antibiotic-free LB broth for up to 50 generations (4 generations per day). The 50th-generation bacterial culture was plated onto MHA plates and incubated overnight at 37°C. A total of 150 single colonies were picked from the MHA plates, and PCR identification was performed to verify the presence of the *bla*_NDM-1_. Several of these colonies were further subjected to sequencing analysis to confirm whether the four tandem *bla*_NDM-1_ structures had undergone any changes.

#### WGS and data analysis

DNA extraction of L710hy was performed using the Genomic DNA Isolation Kit (QIAGEN, Hilden, Germany). Sequencing was performed by Illumina NovaSeq 6000 (Illumina, San Diego, CA, United States) and Oxford Nanopore platform (Oxford Nanopore Technologies, Oxford, United Kingdom). The Illumina short reads and Nanopore long reads were assembled by Unicycler.^23^ Plasmid types were identified by PlasmidFinder.^24^ The search for acquired resistance genes was conducted using ResFinder. BRIG was employed to generate the comparative circular figures.^25^ Genome annotation was performed using RAST.^26^ ISfinder was used to detect genomic mobile elements.^27^ Easyfig generated a comparative image of the genetic environments of the resistance genes.^28^

### Quantification and statistical analysis

There are no quantification or statistical analyses to include in this study.
